# Association between intraoperative heart rate and postoperative myocardial injury in patients following non-cardiac surgery

**DOI:** 10.1186/2197-425X-3-S1-A751

**Published:** 2015-10-01

**Authors:** TEF Abbott, GL Ackland, A Wragg, R Rodseth, A Archbold, RM Pearse

**Affiliations:** William Harvey Research Institute, Queen Mary University of London, London, United Kingdom; University College London, London, United Kingdom; Barts Health NHS Trust, London, United Kingdom; University of KwaZulu-Natal, Durban, South Africa

## Introduction

Approximately 10% patients who undergo non-cardiac surgery show biochemical evidence of myocardial injury, which is associated with cardiac complications and death.[1] The aetiology and mechanisms of perioperative myocardial injury are incompletely understood. Observational studies have described associations between perioperative tachycardia and postoperative cardiac complications. However, 'tachycardia' was often poorly defined and its association with myocardial injury has not been investigated.

## Objectives

The aim of this study was to determine whether or not intraoperative tachycardia is associated with postoperative myocardial injury.

## Methods

This was a post-hoc analysis of an international observational cohort study of patients undergoing non-cardiac surgery.[1] Maximum and minimum intraoperative heart rates were recorded. Postoperative myocardial injury was defined as serum troponin T concentration >0.03 ng/ml within 30 days of surgery. Heart rate was analysed as a continuous variable and then as a categorical variable divided into five groups (< 45, 45-55, 56-100, 101-140, >140 bpm). A multivariable logistic regression model was constructed which included known confounding variables to assess for associations between maximum and minimum intraoperative heart rate and myocardial injury. All-cause mortality within 30 days of surgery was a secondary outcome measure.

## Results

**Figure 1:**
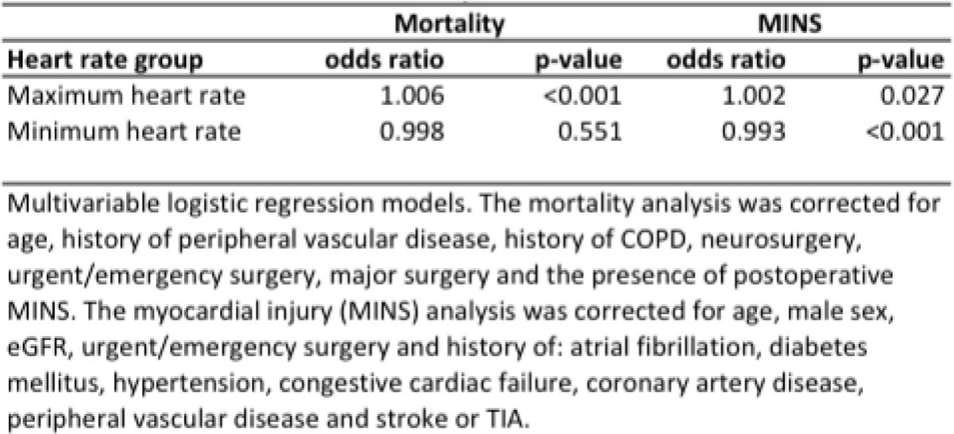
**Maximum and minimum intraoperative heart rate – multivariable model**

1,197 patients (7.9%) of 15,792 patients showed evidence of myocardial injury and 315 (2.0%) died. Maximum intraoperative heart rate was associated with myocardial injury and mortality. Minimum intraoperative heart rate was negatively associated with myocardial injury, but not mortality (figure 1). Maximum intraoperative heart rate >140 bpm was more strongly associated with myocardial injury than heart rate 101-140 (Odds ratios 1.67, p = 0.075 vs, 1.18, p = 0.057; reference group heart rate 55-101 bpm).

## Conclusion

Intraoperative tachycardia was associated with adverse postoperative outcomes. Further work is required to refine the relation between perioperative heart rate and post-operative clinical outcomes and whether or not heart rate modulating drugs reduce perioperative myocardial injury.

## FUNDING

TA is supported by an MRC/BJA Clinical Research Training Fellowship.
